# Species and Phylogenetic Diversity of Woody Plants Shift With the Elevational Gradient in Subtropical Forests in South China

**DOI:** 10.1002/ece3.71761

**Published:** 2025-07-14

**Authors:** Jing Li, Yinghua Luo, Feng Chen, Cong Hu, Chaohao Xu, Zhonghua Zhang, Gang Hu

**Affiliations:** ^1^ Key Laboratory of Environment Change and Resources use in Beibu Gulf, Ministry of Education Nanning Normal University Nanning China; ^2^ Guangxi Key Laboratory of Earth Surface Processes and Intelligent Simulation Nanning Normal University Nanning China; ^3^ Guangxi Key Laboratory of Forest Ecology and Conservation, College of Forestry Guangxi University Nanning China; ^4^ Laibin Jinxiu Dayaoshan Forest Ecosystem Observation and Research Station of Guangxi Laibin China

**Keywords:** elevational gradients, phylogenetic diversity, phylogenetic structure, species diversity, woody plants

## Abstract

The distribution of biodiversity along elevational gradients and the drivers of these patterns are research hotspots in community ecology; nonetheless, these aspects remain insufficiently understood. To address this, we established 24 plots along an elevational gradient from 300 to 1400 m on Daming Mountain, Guangxi, China, and examined the patterns and drivers of species and phylogenetic diversity along this gradient via polynomial regression, generalized linear mixed model, correlation analysis, and redundancy analyses. With increasing elevation, species and phylogenetic diversity showed a hump‐shaped trend, and the phylogenetic structures exhibited clustering at both low and high elevations, whereas at mid‐elevations, a coexistence of clustered and overdispersed structures was observed. Elevation, soil nitrate nitrogen content, and slope collectively constituted the key environmental factors driving the spatial patterns of species diversity. Meanwhile, soil nitrate nitrogen and ammonium nitrogen contents had a decisive influence on phylogenetic diversity. These findings, which reveal the patterns of diversity of woody plant communities along an elevational gradient on Daming Mountain, will contribute to the development of biodiversity conservation strategies for the region.

## Graphical abstract

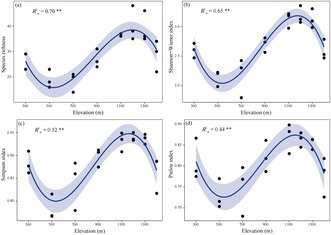

## 1 Introduction

Spatial variation in biodiversity along environmental gradients and its underlying mechanisms are central topics in biogeography and macroecology (Zhang et al. 2022). Understanding spatial patterns and their causes aids in predicting the impacts of global climate change on biodiversity while also informing the development of effective conservation and management strategies (Zhao et al. 2023). In mountain ecosystems, elevational gradients can serve as natural laboratories for studying biodiversity patterns and their underlying mechanisms (Rahbek et al. 2019). In contrast to latitudinal gradients, elevational gradients encompass rapid changes in multiple environmental factors (such as temperature, moisture, and soil) over short geographic distances (Sundqvist et al. 2013), leading to shifts in species composition and diversity along the gradient (Liu 2017). Since Alexander von Humboldt's pioneering work on Mount Chimborazo (von Humboldt 1849), elevational biodiversity gradients have become a central focus in biogeography and ecology (Rahbek 1995; McCain and Grytnes 2010). Despite extensive research, empirical studies reveal substantial variation in diversity patterns across different ecosystems, geographic regions, and taxonomic groups (Peters et al. 2016; Rahbek et al. 2019; Di Biase et al. 2021). This persistent heterogeneity has precluded the emergence of a general theoretical framework for elevational diversity gradients.

The patterns of change in species diversity along elevational gradients vary across regions, increasing with elevation in some regions (Torres et al. 2020), decreasing with elevation in others (Fontana et al. 2020), or displaying a hump‐shaped pattern at mid‐elevations (Kumar et al. 2017). On the basis of these observations, multiple hypotheses have been proposed to address the underlying mechanisms of elevational patterns in diversity, including the water–energy dynamics hypothesis (Huang et al. 2017), the mid‐domain effect hypothesis (Moreno et al. 2008), and the ambient energy hypothesis (Turner 2004). Species distributions vary across geographic regions, taxonomic groups, and sampling scales and are strongly influenced by interactions with many local environmental factors, including microclimatic factors and the intensity of human disturbance (Berhanu et al. 2017; Gebrehiwot et al. 2019). Although many hypotheses to explain elevational patterns have been examined, it remains unclear whether a universal relationship exists between species diversity and elevation (Karel et al. 2011). Therefore, the mechanisms and drivers of species diversity patterns along elevational gradients require further study.

Phylogenetic diversity is an important dimension in community ecology that integrates the phylogenetic and evolutionary relationships among species and reveals information that cannot be captured through species diversity alone (Swenson 2011). A study on the phylogenetic diversity and species richness of plants at the Cape of Good Hope, South Africa, revealed that phylogenetic diversity is a critical metric for assessing the evolutionary history and uniqueness of lineages within specific biogeographical contexts. Applying it to biodiversity conservation decisions can support the development of comprehensive protection strategies for regional communities (Forest et al. 2007; Pio et al. 2011). Environmental filtering and competitive exclusion on the basis of niche theory are currently considered the primary drivers of community assembly processes. Environmental filtering leads to the coexistence in the same habitat of closely related species with similar adaptive traits, resulting in a community structure that exhibits phylogenetic clustering (González‐Caro et al. 2014). Conversely, competitive exclusion prevents closely related species with similar ecological niches from coexisting in the same environment, leading to phylogenetic dispersion within the community (Webb et al. 2006). Environmental factors change sharply along elevational gradients, leading to substantial elevational variation in species and phylogenetic diversity. For instance, Culmsee and Leuschner (2013) analyzed tree species diversity along an elevational gradient in the primary forests of the Malay Archipelago, finding that species richness decreased, whereas phylogenetic diversity increased, with increasing elevation. Zhang et al. (2021) studied community assembly drivers along an elevational gradient in the subtropical forests of eastern China, observing that species diversity increased with elevation, whereas phylogenetic structure did not change notably. Conversely, for the subtropical Nujiang Valley of southwestern China, Yue and Li (2021) found that both species richness and phylogenetic diversity declined significantly with increasing elevation, with phylogenetic clustering at higher elevations. Patterns of phylogenetic diversity along elevational gradients thus vary among regions.

Plant diversity is closely linked to soil environmental factors (Hooper et al. 2012), which directly determines the availability of essential elements for plant growth, thus influencing species diversity within plant communities (Crawford and Rudgers 2012). Soil environmental factors affect forest‐community species composition and distribution by regulating the nutrient supply (Bin et al. 2016). Interactions between plants and soil are key drivers of ecosystem evolution (Liu et al. 2021), with the forest‐community structure and species composition affecting soil nutrient levels. Feng et al. (2023) found that differences in plant communities and litter resulted in higher soil‐nutrient levels at mid‐elevations than at high and low elevations, indirectly limiting species diversity in aboveground communities. However, Song et al. (2020) found that higher elevational zones with greater precipitation and higher temperatures exhibit greater organic matter decomposition; high‐elevation mountain ecosystem soils thus exhibit better nutrient retention, providing more nutrients for plant growth and thus supporting greater species diversity within communities. For forests in the Amazon Basin, Quesada et al. (2012) found that soil nutrient availability promoted rapid plant growth, leading to increased aboveground biomass and positively affecting species diversity. Conversely, for the northern subtropical‐warm temperate transition zone in China, Yu et al. (2023) found that soil nutrients facilitated the growth of dominant species, which exhibited significant competitive advantages, leading to the exclusion of other species and thus reducing community species diversity. Soil environmental factors, therefore, play crucial roles in plant distribution and the formation of community structure.

Evergreen broadleaf forests are the most representative vegetation type in the subtropical mountainous ecosystems of China. As a major component of global evergreen broadleaf forests, they boast rich plant diversity and endemism and serve as critical areas for ecosystem services (Liu et al. 2018; Ouyang et al. 2019). Daming Mountain, located in the central‐southern region of Guangxi, near the Tropic of Cancer, exhibits characteristics of a South Asian subtropical monsoon climate. It hosts several vegetation types along its elevational gradient, with Subtropical Monsoon Evergreen Broadleaf Forest, the zonal vegetation type, serving as a genetic reservoir of zonal biological species and a laboratory for observing ecosystem balance (Li et al. 2023). To analyze the patterns and drivers of species and phylogenetic diversity along the elevation in this region, we selected forest plots at different elevations. The objectives of the present study were to: (1) clarify the elevational distribution patterns of species diversity and phylogenetic diversity; and (2) reveal the influencing factors on the elevational distribution of species and phylogenetic diversity. These findings provide a theoretical basis for biodiversity conservation in mountain ecosystems and ecological restoration of forest ecosystems.

## 2 Materials and Methods

### 2.1 Study Area

Daming Mountain, in the south‐central region of Guangxi, South China (23°24′‐23°30′N, 108°20′‐108°34′ E), belongs to the South Subtropical Monsoon Climate Zone. The average annual temperature is 15.1°C, with a mean temperature of 21.9°C in July and 5.8°C in January. The average annual precipitation is 2630 mm, with elevation ranging from 115 m to 1760 m, and an average elevation of 1200 m. Daming Mountain's unique geographical position, ancient geological strata, and developed landforms contribute to its rich biodiversity. The area preserves relatively intact primary forests, making it a significant natural biological repository (Wang et al. 2016). Its diverse and complex vegetation types, which result from the combined influences of factors such as elevation, soil, climate, and moisture, include South Subtropical Monsoon Evergreen Broadleaf Forests, the zonal type, as well as Montane Evergreen Broadleaf Forests, Evergreen‐Deciduous Broadleaf Mixed Forests, Coniferous Forests, Mixed Broadleaf‐Coniferous Forests, and Mossy Dwarf Forests at the summit (Li et al. 2023).

### 2.2 Plot Survey

**FIGURE 1:**
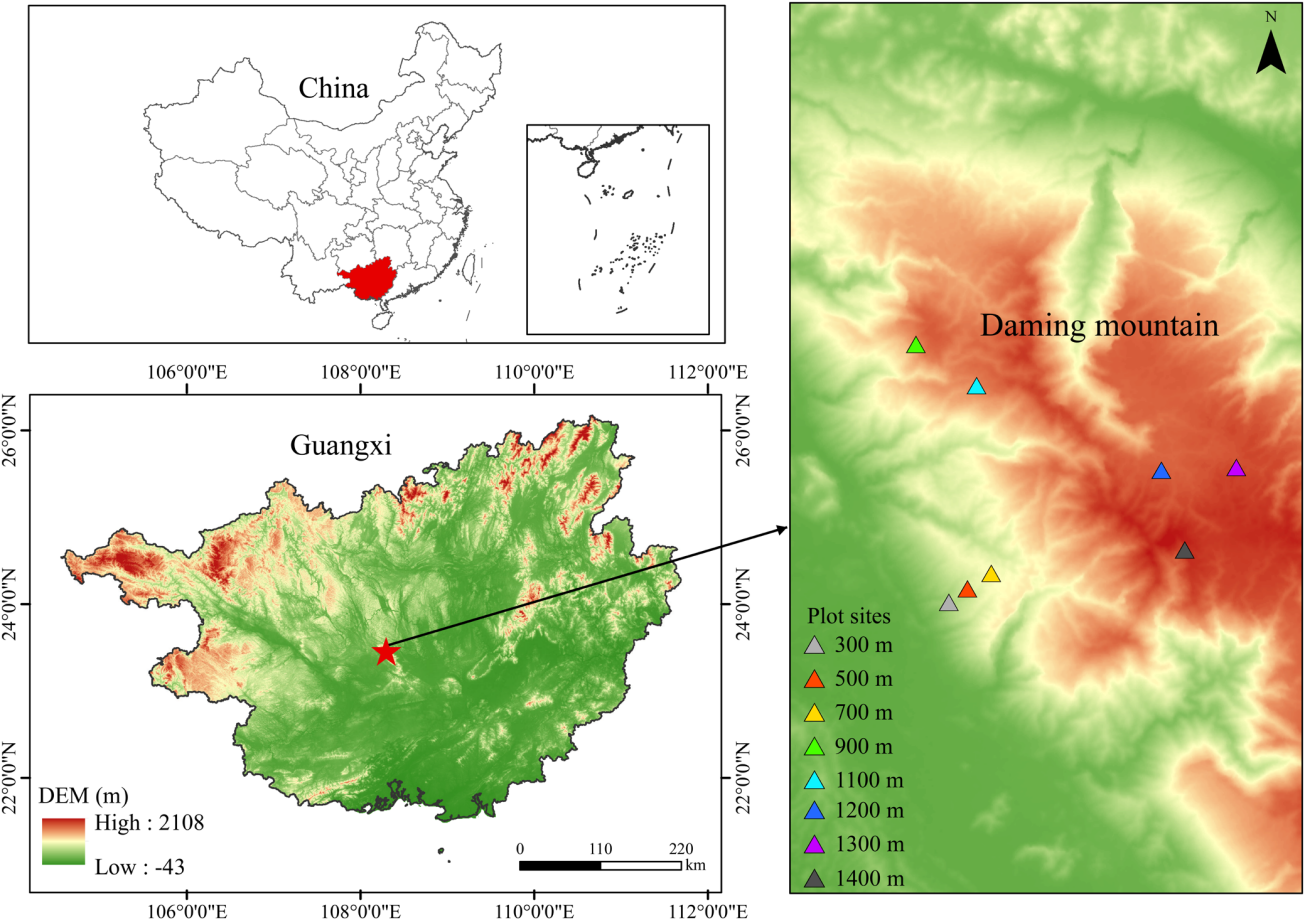
Location of the study area and plots.

In 2021, we established a total of 24 permanent forest plots (20 × 20 m each) along an elevational gradient on Daming Mountain, spanning eight elevations (300, 500, 700, 900, 1100, 1200, 1300, and 1400 m a.s.l.), with three replicate plots per elevation (Figure 1). Within each plot, we censused all woody plants with a diameter at breast height (DBH) ≥ 1 cm. DBH and tree height were measured using standardized field protocols, with diameter tapes and telescopic height poles. Each individual was tagged with a unique identifier to enable long‐term monitoring. To quantify canopy structure, we captured hemispherical photographs at the center of each plot using a fisheye lens‐equipped digital camera. Canopy closure was subsequently analyzed using HemiView 2.1.1 software. Geographic coordinates (latitude and longitude), elevation, and slope were recorded for each plot using a high‐precision GPS unit (Table 1).

**TABLE 1:** Basic information about each forest plot.

Elevation (m)	Coordinates	Slope (°)	Canopy density	Dominant species
300	108.37979° E	24.67 ± 2.85 abc	0.81 ± 0.02 a	*Lindera metcalfiana*
	23.46555° N			*Microdesmis caseariifolia*
				*Sloanea leptocarpa*
500	108.38449° E	28.33 ± 8.82 ab	0.79 ± 0.04 a	*Clethra bodinieri*
	23.46933° N			*Engelhardia roxburghiana*
				*Symplocos lancifolia*
700	108.39098° E	16.67 ± 2.40 abcd	0.78 ± 0.04 a	*Rhodoleia championii*
	23.47313° N			*Rhododendron moulmainense*
				*Clethra bodinieri*
900	108.37332° E	35.00 ± 2.89 a	0.80 ± 0.03 a	*Rhodoleia championii*
	23.52587° N			*Machilus thunbergii*
				*Rhododendron cavaleriei*
1100	108.38845° E	17.67 ± 1.86 abcd	0.85 ± 0.04 a	*Castanopsis fargesii*
	23.51631° N			*Rhododendron cavaleriei*
				*Camellia fraterna*
1200	108.43393° E	11.67 ± 2.19 bcd	0.86 ± 0.01 a	*Castanopsis eyrei*
	23.49639° N			*Litsea elongata*
				*Engelhardia roxburghiana*
1300	108.45266° E	2.33 ± 0.33 d	0.81 ± 0.01 a	*Engelhardia roxburghiana*
	23.49602° N			*Machilus thunbergii*
				*Schima argentea*
1400	108.43930° E	6.00 ± 1.15 cd	0.81 ± 0.01 a	*Camellia fraterna*
	23.47731° N			*Michelia maudiae*
				*Symplocos sumuntia*

*Note:* Results are presented as mean ± standard error. Different lowercase letters in the same column represent significant differences between elevational gradients (*p* < 0.05).

### 2.3 Environmental Variables

In each plot, we collected soil samples using the five‐point sampling method. After removing the surface litter, we used a cutting ring to collect topsoil from a depth of 0–20 cm and collected an additional 100 g of soil around the ring. The samples from the five points were thoroughly mixed and taken to the laboratory. We recorded the fresh mass of the soil samples from the cutting ring, then dried and weighed them in the laboratory to calculate soil water content. The fresh soil was air‐dried and passed through a 2 mm sieve for chemical analysis. Soil organic carbon was measured using the external–heating potassium dichromate oxidation method, total nitrogen using the Kjeldahl method, total phosphorus via sodium hydroxide fusion–molybdenum antimony anti‐colorimetry, total potassium via flame atomic absorption spectrometry, available phosphorus via sodium bicarbonate extraction–molybdenum antimony anti‐spectrophotometry, nitrate nitrogen via dual–wavelength ultraviolet spectrophotometry, ammonium nitrogen via extraction–indophenol blue colorimetry, and pH using an electrode.

### 2.4 Data Analysis

#### 2.4.1 Species Diversity

On the basis of the species matrices obtained from the plot survey, we selected the Species richness index (*R*, the total number of species present in the plot), Shannon–Wiener index (*H*), Simpson index (*D*), and Pielou index (*J*) (Hill 1973) to evaluate differences in species diversity along the elevational gradient. Calculations were performed using the ‘vegan’ package in R 4.0.3, as follows:

$$H=-\sum_{i=1}^{s}P_{i}\ln\left(P_{i}\right)$$

$$D=1-\sum_{i=1}^{s}{P_{i}}^{2}P_{i}=N_{i}/N$$

J=H/lnS

where *Ni* denotes the importance value of species *i* in the plot and *N* is the sum of the importance values of all species in the plot.

#### 2.4.2 Phylogenetic Diversity and Structure

We used the ‘plantlist’ package in R 4.0.3 to retrieve the family, genus, and species information for each recorded species. The ‘V.phyloMaker 2’ package (Jin and Qian 2019; Qian and Jin 2016) was utilized with the phylo.maker function to construct the super phylogenetic tree for this study. The ‘picante’ package was then used to calculate the phylogenetic diversity (PD) and to characterize the phylogenetic structure using the net relatedness index (NRI) and nearest taxon index (NTI). The phylogenetic structure index was calculated by synthesizing the super phylogenetic tree from the community species pool and comparing each actual community with a standardized null model, and differences between the observed values and those expected under the null hypothesis were measured. The null model was created by randomly shuffling the terminal branches of the evolutionary tree 999 times, ensuring that each null model and generated phylogenetic tree maintained the same number of species. NTI, which is used to evaluate the phylogenetic structure of the communities from the terminal branches, is significantly influenced by polytomies at these tips; NRI, in contrast, is used to assess the overall community phylogenetic structure (Kembel et al. 2010), as follows:

$$\mathrm{PD}=\Sigma L_{b}$$

$$\mathrm{NRI}=-1\times \frac{\mathrm{MP}D_{S}-\mathrm{MP}D_{\mathrm{mds}}}{\mathrm{SD}\left(\mathrm{MP}D_{\mathrm{mds}}\right)}$$

$$\mathrm{NTI}=-1\times \frac{\mathrm{MNT}D_{S}-\mathrm{MNT}D_{\mathrm{mds}}}{\mathrm{SD}\left(\mathrm{MNT}D_{\mathrm{mds}}\right)}$$

where Lb represents the branch length of the phylogenetic tree and MPD*s* and MNTDs are the observed mean pairwise distance (MPD) and mean nearest taxon phylogenetic distance (MNTD) within the community, respectively. MPDmds and MNTDmds represent the mean MPD and MNTD for the 999 randomized communities under the null model, and SD refers to standard deviation. A positive NRI or NTI indicates phylogenetic clustering, whereas a negative NRI or NTI indicates phylogenetic overdispersion. An NRI or NTI of zero indicates that the plot phylogenetic structure is random (Webb et al. 2008).

### 2.5 Statistical Analysis

To quantify elevational patterns of taxonomic and phylogenetic diversity, we fitted generalized linear mixed models (GLMMs) with elevation as the primary predictor. Species richness was modeled using a Poisson distribution, whereas all other response variables followed a Gaussian distribution (Luo et al. 2023). Elevation was treated as a fixed effect, and plots were treated as random effects in the model to estimate parameters and explanatory power. The marginal *R* ^*2*^ (*R* ^*2*^_*m*_) represents the variance explained by fixed effects only, whereas the conditional R^2^ includes both fixed and random effects (Nakagawa and Schielzeth 2013). Given the potential nonlinearity in biodiversity variation along the elevational gradient, we fitted linear, quadratic, and cubic models and selected the model with the lowest AIC as the optimal one. The Shapiro–Wilk test of normality and a homogeneity of variance test were used to examine changes in environmental factors across the elevational gradient. For data meeting the assumptions of normality and homogeneous variance, one‐way ANOVA followed by the Least Significant Difference (LSD) test for pairwise comparisons was applied. For data not meeting these assumptions, the Kruskal–Wallis test with pairwise tests was applied. Differences were considered significant at *p* < 0.05. Pearson correlation analysis was used to examine the relationships between species diversity, phylogenetic diversity, and environmental factors. Additionally, redundancy analysis (RDA) was conducted to further explore the influence of environmental factors on species and phylogenetic diversity, and the explanatory power of each environmental variable was calculated. All statistical analyses were performed using R software (version 4.0.3) with the packages “psych” (Revelle 2023), “vegan” (Oksanen et al. 2022), “rdacca.hp” (Lai et al. 2022), and “MuMIn” (Barton 2019).

## 3 Results

### 3.1 Elevational Changes in Species and Phylogenetic Diversity

**FIGURE 2:**
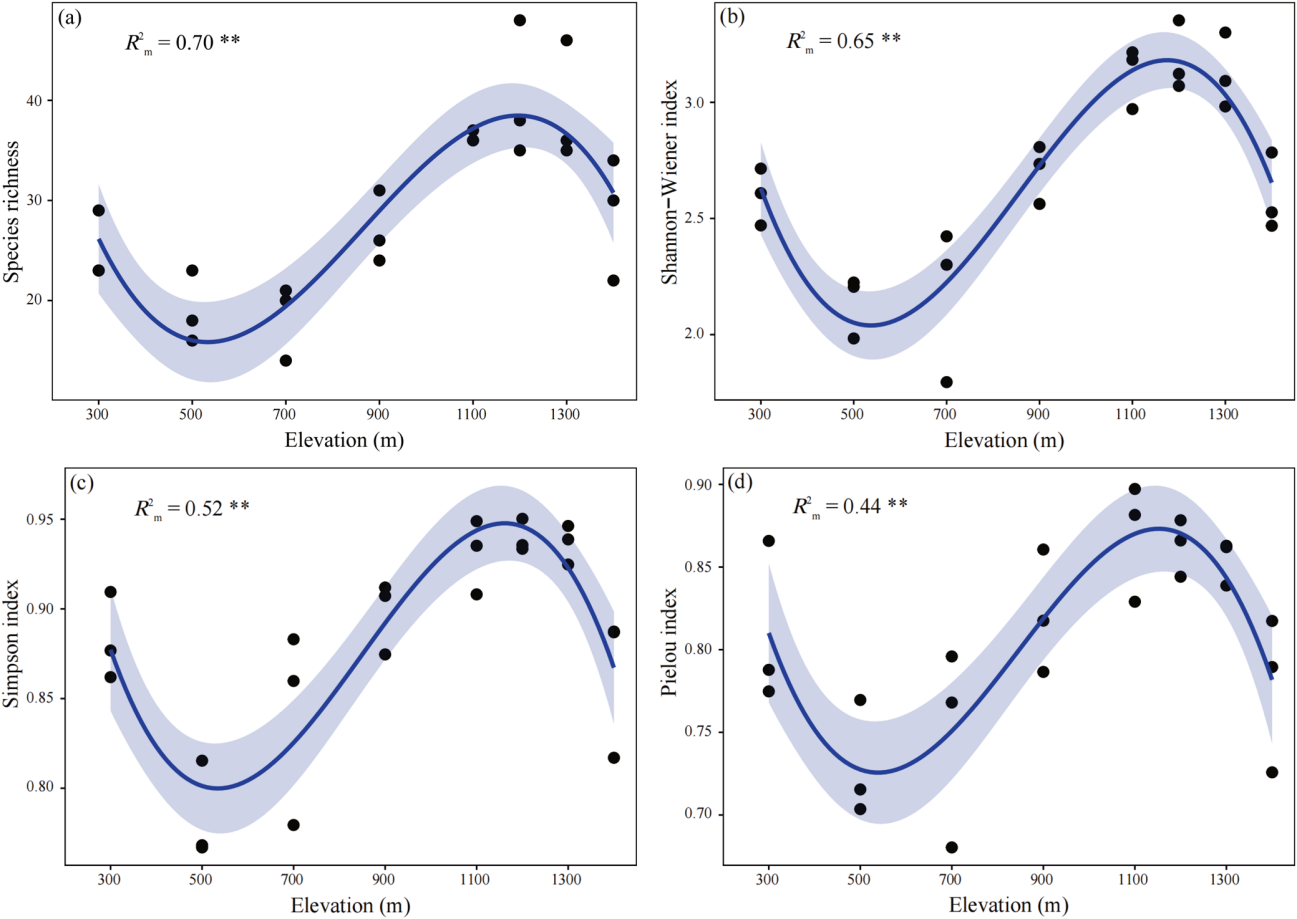
Changes in the species richness (a), Shannon–Wiener (b), Simpson (c), and Pielou (d) indices with elevational gradient. **p < 0.01.

The Species richness, Shannon–Wiener, Simpson, and Pielou indices show a hump‐shaped trend along the elevational gradient, first decreasing, then increasing, and finally decreasing (Figure 2, *R* ^2^_m_ = 0.70, *R* ^2^_m_ = 65, *R* ^2^_m_ = 52, *R* ^2^_m_ = 0.44), with minima at 700 m for Species richness, Shannon–Wiener, and Pielou indices (Figure 2a,b,d) and peaks at 1200 m for species richness, Shannon–Wiener, and Simpson indices.

**FIGURE 3:**
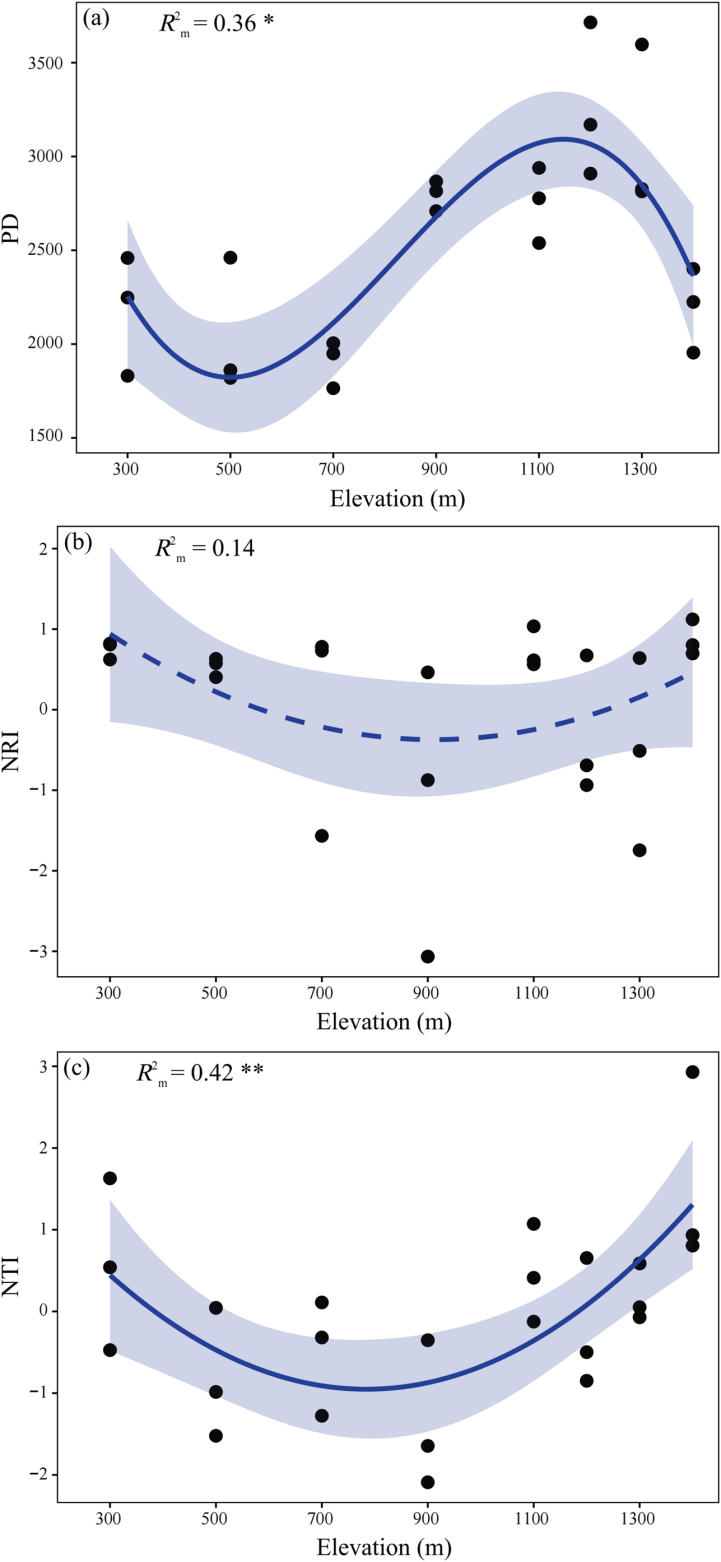
Changes in phylogenetic diversity (PD) (a), net relatedness index (NRI) (b), and nearest taxon index (NTI) (c) with elevational gradient. *p < 0.05, **p < 0.01.

PD also exhibited a hump‐shaped trend with increasing elevation (Figure 3a, *R* ^2^_m_ = 0.36). However, the trend in NRI with elevation was not clear (Figure 3b, *R* ^2^_m_ = 0.14). NTI displayed an inverted hump‐shaped trend along the elevational gradient (Figure 3c, *R* ^2^_m_ = 0.42). At both low and high elevations, most of the plots exhibited NRI and NTI values significantly greater than zero, indicating phylogenetic clustering. At mid‐elevations, two types of phylogenetic community structures were observed, some clustered and others overdispersed.

### 3.2 Elevational Changes in Soil Physical and Chemical Properties

Soil organic carbon and total nitrogen first increased and then declined with increasing elevation, both peaking at 1200 m, at 141.15 and 6.87 g·kg^−1^, respectively, although these changes were not statistically significant (*p* > 0.05) (Table 2). Total potassium and total phosphorus exhibited no clear trends with elevation. Total potassium peaked at 35.61 g·kg^−1^ at 700 m and was lowest at 1400 m, varying significantly between 700 m and 1400 m (*p* < 0.05). Total phosphorus peaked at 1200 m at 0.63 g·kg^−1^ and was lowest at 1300 m, varying significantly between these elevations (*p* < 0.05). Available phosphorus and nitrate nitrogen peaked at mid‐to‐high elevations. Available phosphorus peaked at 21.19 mg·kg^−1^ at 1200 m and was lowest at 700 m, varying significantly between these elevations (*p* < 0.05). Nitrate nitrogen peaked at 47.12 mg·kg^−1^ at 1100 m and was lowest at 700 m, varying significantly between these elevations (*p* < 0.05). Ammonium nitrogen, soil water content, and soil pH all peaked at 1400 m but showed no significant variation with elevation (*p* > 0.05).

**TABLE 2:** Changes in physicochemical properties of soils at different elevational gradients.

Elevation (m)	SOC (g·kg^−1^)	TN (g·kg^−1^)	TK (g·kg^−1^)	TP (g·kg^−1^)	AP (mg·kg^−1^)	NN (mg·kg^−1^)	AN (mg·kg^−1^)	SWC (%)	pH
300	25.86 ± 4.29 a	2.28 ± 0.34 a	23.30 ± 2.22 abc	0.35 ± 0.05 ab	1.36 ± 0.22 b	26.21 ± 1.92 ab	9.11 ± 6.64 a	22.87 ± 1.24 a	3.42 ± 0.04 a
500	43.82 ± 7.84 a	2.76 ± 0.23 a	21.14 ± 3.38 abc	0.30 ± 0.02 ab	1.29 ± 0.14 b	10.18 ± 3.43 bc	9.76 ± 3.69 a	40.82 ± 1.50 a	3.61 ± 0.04 a
700	44.73 ± 9.21 a	3.01 ± 0.48 a	35.61 ± 2.40 a	0.30 ± 0.01 ab	0.76 ± 0.15 b	1.14 ± 0.58 c	19.85 ± 4.12 a	47.14 ± 3.06 a	3.69 ± 0.03 a
900	45.14 ± 11.53 a	3.01 ± 0.71 a	26.75 ± 2.17 ab	0.49 ± 0.11 ab	1.58 ± 0.27 b	28.41 ± 3.43 ab	3.35 ± 0.96 a	47.64 ± 12.42 a	3.64 ± 0.08 a
1100	52.00 ± 17.76 a	2.71 ± 1.22 a	12.15 ± 3.76 bc	0.37 ± 0.05 ab	3.93 ± 1.59 ab	47.12 ± 7.46 a	32.29 ± 10 a	70.46 ± 14.23 a	3.51 ± 0.10 a
1200	141.15 ± 80.26 a	6.87 ± 3.64 a	19.87 ± 6.02 abc	0.63 ± 0.13 a	21.19 ± 8.44 a	46 ± 3.39 a	69.9 ± 26.15 a	68.41 ± 5.54 a	3.48 ± 0.22 a
1300	46.84 ± 3.64 a	2.81 ± 0.24 a	13.72 ± 1.74 bc	0.24 ± 0.02 b	7.76 ± 1.89 ab	8.12 ± 0.13 bc	54.66 ± 20.33 a	54.92 ± 5.46 a	3.67 ± 0.04 a
1400	65.66 ± 28.87 a	3.48 ± 0.79 a	7.74 ± 0.54 c	0.32 ± 0.04 ab	16.64 ± 5.85 ab	44.45 ± 10.12 a	80.2 ± 52.53 a	75.62 ± 27.64 a	3.85 ± 0.05 a

*Note:* Results are presented as mean ± standard error. Different lowercase letters in the same column represent significant differences between elevational gradients (*p* < 0.05).

Abbreviations: AN, ammonium nitrogen; AP, available phosphorus; NN, nitrate nitrogen; SOC, soil organic carbon; SWC, soil water content; TK, total potassium; TN, total nitrogen; TP, total phosphorus.

### 3.3 Environmental Drivers of Elevational Variation in Species and Phylogenetic Diversity

**FIGURE 4:**
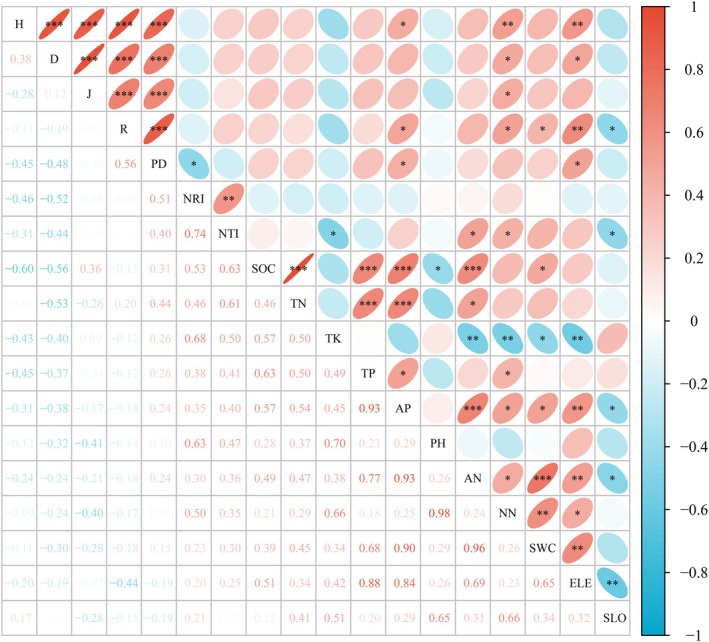
Correlation analysis relating plant species diversity, phylogenetic diversity, and environmental variables. AN, ammonium nitrogen; AP, available phosphorus; D, Simpson index; ELE, elevation; H, Shannon–Wiener index; J, Pielou index; NN, nitrate nitrogen; NRI, net relatedness index; NTI, nearest taxon index; PD, phylogenetic diversity; R, species richness index; SLO, slope; SOC, soil organic carbon; SWC, soil water content; TK, total potassium; TN, total nitrogen; TP, total phosphorus. *p < 0.05, **p < 0.01, ***p < 0.001.

The Shannon–Wiener index was positively correlated with nitrate nitrogen, elevation, and available phosphorus (*p* < 0.05) (Figure 4). The Simpson index was positively correlated with soil nitrate nitrogen and elevation (*p* < 0.05). The Pielou index was positively correlated with soil nitrate nitrogen (*p* < 0.05). The Species richness index was positively correlated with elevation, soil available phosphorus, nitrate nitrogen, and moisture content (*p* < 0.05), whereas it was negatively correlated with slope (*p* < 0.05). PD was positively correlated with available phosphorus (*p* < 0.05). NTI was positively correlated with soil ammonium nitrogen and nitrate nitrogen (*p* < 0.05) and negatively correlated with total potassium and slope (*p* < 0.05).

**FIGURE 5:**
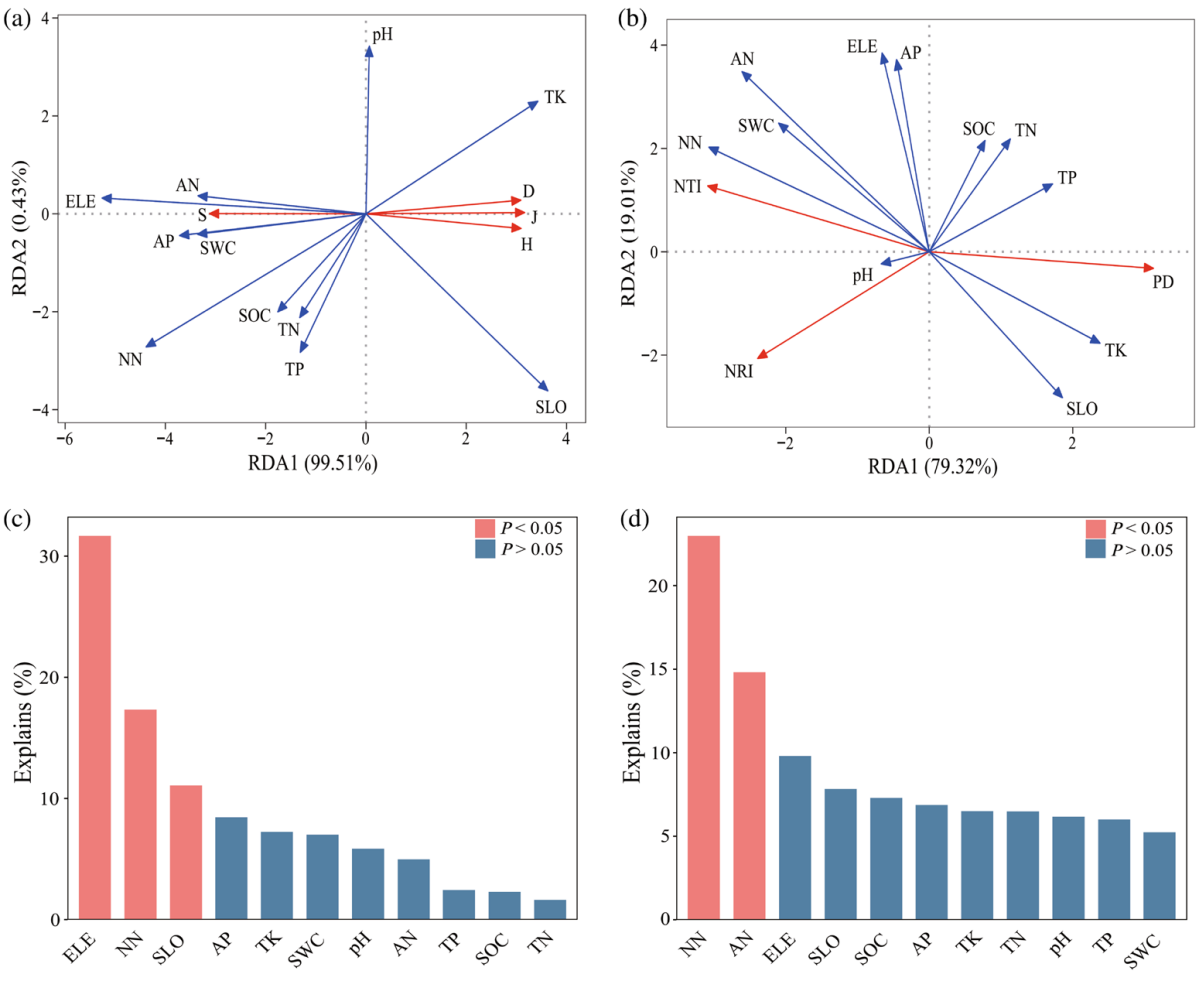
Redundancy analysis of plant species diversity (a), phylogenetic diversity (b), and environmental variables. The explanatory rate of environmental variables on species diversity (c) and phylogenetic diversity (d). AN, ammonium nitrogen; AP, available phosphorus; D, Simpson index; ELE, elevation; H, Shannon–Wiener index; J, Pielou index; NN, nitrate nitrogen; NRI, net relatedness index; NTI, net nearest taxa index; PD, phylogenetic diversity index; R, Species richness index; SLO, slope; SOC, soil organic carbon; SWC, soil water content; TK, total potassium; TN, total nitrogen; TP, total phosphorus. The red line indicates the response variable, and the blue line indicates the explanatory variable.

The RDA results showed that RDA1 and RDA2 explained 99.51% and 0.43% of the variance in species diversity, respectively (Figure 5a). Elevation, soil nitrate nitrogen content, and slope were identified as the primary influencing factors, explaining 31.7%, 17.3%, and 11.1% of the variance, respectively (Figure 5c). For phylogenetic diversity, RDA1 and RDA2 accounted for 79.32% and 19.01% of the variance, respectively (Figure 5b). Soil nitrate nitrogen and ammonium nitrogen contents were the main influencing factors, explaining 23.0% and 14.9% of the variance, respectively (Figure 5d).

## 4 Discussion

### 4.1 Species Diversity and Phylogenetic Diversity Along an Elevational Gradient

Elevation serves as a key driver of plant community assembly by mediating critical environmental gradients, including solar radiation, temperature, moisture availability, and soil properties. These elevational controls regulate both ecosystem‐level hydrological processes and species‐specific physiological constraints, ultimately governing the spatial organization of plant biodiversity across mountain landscapes (Zhu et al. 2019). Here, species diversity and phylogenetic diversity had a hump‐shaped trend along the elevation gradient, peaking at 1200 m. Similar patterns have been observed in other regions, such as central Veracruz in Mexico (Gómez‐Díaz et al. 2017), the northwestern Himalayas of India (Sharma and Kala 2022), and on Mount Kenya (Zhou et al. 2019). The higher plant diversity recorded at mid‐high elevations is attributed to the optimal climatic conditions at these elevations that allow for the coexistence of many species. McCain (2007) proposed a climate model in which the temperature and precipitation at middle elevations promote higher plant diversity. Our findings indicate that mid‐elevation zones on Daming Mountain exhibit optimal hydrothermal conditions (Huang et al. 2015; Li et al. 2023), creating favorable habitats that support higher plant species richness. This pattern aligns with the ecotone hypothesis, where transitional elevations act as biodiversity hotspots because of the overlap of distinct plant communities (Niu et al. 2024) and increased environmental heterogeneity that facilitates species coexistence (Chen et al. 2025). The elevational diversity gradient is further modulated by anthropogenic influences. At lower elevations, intensive human activities, including selective logging, economic plantations, and tourism development, have significantly reduced species diversity through habitat degradation and local extirpations (Manzoor et al. 2025). In contrast, higher elevations experience minimal disturbance, allowing species richness to increase with elevation (Zhang et al. 2016). This dual control of environmental filtering and human pressure underscores the complex drivers of biodiversity patterns in montane ecosystems. Species richness, Shannon–Wiener, and Pielou indices were all lowest at 700 m. The initial decline in diversity may be linked to the significant human disturbance in this zone, as reflected by the presence of plantations of economic trees such as *Illicium verum* (Li et al. 2023).

Habitat filtering and competitive exclusion are opposing processes that promote species coexistence. Habitat filtering leads to phylogenetic clustering, whereas competitive exclusion prevents closely related species from coexisting, resulting in phylogenetic dispersion (Bracewell et al. 2017; Li et al. 2018). Here, the forest communities exhibited phylogenetic clustering at low elevations, indicating that habitat filtering was predominant at these elevations. Two explanations may account for this. First, temperature and moisture exert substantially less influence at the lower elevations, with the lower topographic and habitat heterogeneity facilitating the coexistence of evolutionary similar lineages (Bryant et al. 2008). Second, human disturbance may act as an environmental filter at lower elevations, thus promoting phylogenetic clustering (Helmus et al. 2010). At mid‐elevations, both dispersed and clustered phylogenetic patterns emerged, which we attribute to the greater habitat heterogeneity at these elevations. This complexity likely promotes different resource utilization strategies among species, thus intensifying interspecific competition, causing both competitive exclusion and habitat filtering to operate in this elevational zone (Li et al. 2022). The clustering at high elevations was likely driven by environmental filtering, and particularly by climatic factors (Qian et al. 2014; Li et al. 2014). At high elevations, most species are physiologically constrained by the harsh climatic conditions, such as low winter temperatures. This strong habitat filtering excludes species that are not adapted to the cold, leaving only a few hardy lineages present and thus causing phylogenetic clustering (Hawkins et al. 2014; Wiens and Donoghue 2005).

### 4.2 Changes in Soil Factors Across Different Elevational Gradients

Soil, a key medium for interactions between organisms and their environment, stores significant amounts of nutrients such as carbon, nitrogen, and phosphorus. These nutrients support vegetation growth and development, thereby influencing plant community structure and function (Verdú et al. 2018). In nutrient‐rich microenvironments, plants can selectively alter root growth to enhance nutrient uptake. Soil nutrient levels directly impact community productivity, with richer soil nutrients leading to higher productivity within the community (Zhang, Gao, et al. 2018). Here, soil organic carbon and total nitrogen exhibited a unimodal pattern, peaking at 1200 m. This is potentially because of the lower temperatures at higher altitudes, which slow biogeochemical processes such as carbon cycling (Bangroo et al. 2017). As elevation increases, soil temperature decreases and moisture increases, reducing the activity of soil fauna and microbes, thus slowing organic matter decomposition and limiting organic carbon and nitrogen mineralization, whereas increasing organic compound input, and causing soil organic carbon and total nitrogen to be elevated at higher elevations (Ren et al. 2017). The lower temperatures at high elevations affect water viscosity and membrane permeability, further inhibiting microbial activity and ultimately reducing organic carbon and total nitrogen bioavailability (Reich and Oleksyn 2004). Total phosphorus and total potassium content did not vary significantly with elevation because, unlike carbon and nitrogen—which primarily come from plant photosynthesis and atmospheric deposition—phosphorus and potassium mainly originate from rock weathering. Phosphorus and potassium in the soil and biosphere exhibit relatively closed and slow cycles (Li et al. 2017). Available phosphorus, nitrate nitrogen, and ammonium nitrogen peaked at mid‐to‐high elevations, possibly owing to an increase in soil moisture, plant litter, and organic matter content with elevation. These conditions provide more nutrients for microbial growth, promoting microbial proliferation and activity, thereby enhancing the efficiency and rate of organic matter utilization and mineralization and ultimately improving soil nutrient status (Pingree and DeLuca 2018; Zhang, Qiao, et al. 2018).

### 4.3 Drivers of Species and Phylogenetic Diversity

The heterogeneity of mountainous topography drives microenvironmental differentiation through light‐thermal and hydrological processes, thereby directly filtering plant adaptability and regulating community assembly, ultimately shaping the spatial patterns of vegetation and biodiversity (Davies et al. 2007). The redundancy analysis in this study indicates that elevation and slope are key environmental factors influencing species diversity. Brambach et al. (2017) found in their study of tropical montane forests in Indonesia that the elevation gradient explained 50% of the variance in species diversity and that elevation changes significantly affect soil nutrient content. In addition, slope, as an important topographic factor, influences the distribution patterns of plant diversity by regulating solar radiation interception, water infiltration, and soil retention capacity (Sherman et al. 2008). This study found that elevation not only directly affects plant diversity but also indirectly shapes community structure by regulating soil nutrients. The Shannon–Wiener and Species richness indices and phylogenetic diversity were significantly correlated with available phosphorus, consistent with the findings of Xu et al. (2016), who identified available phosphorus as a dominant driver of plant diversity in tropical seasonal rainforests. Available phosphorus is crucial for plant growth and development, and its deficiency can filter out species that are not adapted to low‐phosphorus conditions, thereby restricting plant growth and distribution (Merunková and Chytrý 2012). Soil nitrate nitrogen was significantly correlated with the Simpson index and NTI, consistent with the results of Grace et al. (2016). A lack of nitrogen in the soil can hinder photosynthesis, affecting plant growth, reducing differences among the ecological niches, leading to trait convergence, reducing functional and phylogenetic diversity, and affecting species diversity. NTI was significantly correlated with soil total potassium, consistent with the findings of Gartlan et al. (1986), who reported a notable correlation between soil potassium and species diversity in tropical plant communities. Plant absorption of available potassium can influence leaf area and stomatal conductance, thereby regulating photosynthesis and respiration and impacting plant development, metabolism, and morphology, ultimately indirectly limiting plant growth and distribution (Sardans and Peñuelas 2021). Overall, the observed plant diversity patterns emerge from complex interactions among multiple environmental drivers. Although macroclimatic variables (e.g., temperature and precipitation regimes) are known to exert fundamental constraints on plant diversity (Currie et al. 2004), our current analysis focused primarily on topographic and edaphic factors. Future research should incorporate high‐resolution climatic data to quantitatively assess the relative importance of climate versus local environmental filters and the potential climate‐mediated mechanisms underlying diversity patterns across elevational gradients.

## 5 Conclusion

The species and phylogenetic diversity of Daming Mountain exhibited a hump‐shaped trend with increasing elevation; the phylogenetic structure showed clustering at both low and high elevations, whereas at mid‐elevations, both clustered and overdispersed structures coexisted. Soil organic carbon and total nitrogen content initially increased and then decreased with elevation, whereas total potassium and phosphorus did not vary significantly with gradient. Available phosphorus, nitrate nitrogen, ammonium nitrogen, soil water content, and soil pH all peaked at mid‐to‐high elevations. The Shannon–Wiener index, species richness, and phylogenetic diversity were closely associated with soil available phosphorus content, whereas nitrate nitrogen content significantly influenced both species diversity and the nearest taxon index. Elevation and slope are the main environmental factors influencing changes in species diversity, whereas soil factors are the key drivers affecting both species and phylogenetic diversity. Since elevation encompasses multiple environmental variables, future studies could benefit from investigating the effects of temperature, precipitation, and light intensity, as well as incorporating a broader range of abiotic factors. Overall, our study provides valuable theoretical insights for the conservation and management of mountain forest ecosystems, establishing a foundation for biodiversity preservation in forested regions.

## Author Contributions

**Jing Li:** conceptualization (equal), data curation (equal), methodology (equal), writing – original draft (equal). **Yinghua Luo:** funding acquisition (equal), supervision (equal). **Feng Chen:** visualization (equal). **Cong Hu:** methodology (equal). **Chaohao Xu:** methodology (equal). **Zhonghua Zhang:** project administration (equal), supervision (equal), visualization (equal), writing – review and editing (equal). **Gang Hu:** conceptualization (equal), funding acquisition (equal), supervision (equal), visualization (equal), writing – review and editing (equal).

## Conflicts of Interest

The authors declare no conflicts of interest.

## Supporting information


Appendix S1.

Appendix S2.

Appendix S3.

Appendix S4.

## Data Availability

I confirm that the Data Availability Statement is included in the main file of my submission, and that access to all necessary data files is provided to editors and reviewers.
